# Phenolic Compounds as Potential Antioxidant

**DOI:** 10.17795/jjnpp-15380

**Published:** 2013-11-02

**Authors:** Nahid Pourreza

**Affiliations:** 1 Department of Chemistry, College of Science, Shahid Chamran University, Ahvaz, IR Iran

**Keywords:** Antioxidants, Honey

An antioxidant may be roughly defined as a substance that when present at low concentrations, lower than the oxidizable compound to be protected, significantly delays or inhibits its oxidation. There are two basic categories of antioxidants, natural and synthetic, the second ones have been found to cause long-term toxicological effects, including carcinogenicity (1). Attentions have been paid to both natural and synthetic antioxidants. Antioxidants have been intensively studied over the past few decades. Antioxidants play a vital role against the deteriorating action of free radicals in the organisms. Deficiency of antioxidants in living organisms leads to oxidative stress. It has been found that antioxidant compounds present in food, and biological systems are fundamental to the protection of biomolecules from these free-radical redox reactions.

There has been a great interest in phenolic compounds and their antioxidant activity among consumers and the scientific community in the past decade because of the epidemiological studies linking the consumption of diets rich in natural antioxidants with decreased risk of diseases associated with oxidative stress, such as cancer and cardiovascular disease (2). Phenolic compounds as bioactive substances are a large heterogeneous group of secondary plant metabolites that have been widely distributed in plants and are important constituents of human diet (3). The natural products rich in antioxidants are of great importance for scientists. For this reason antioxidant and antitumor activities of sulfated polysaccharide isolated from marine algae have been studied by many scientists. Marine resources have attracted a great attention in the search for bioactive substance to develop new drugs and healthy foods, because of their relatively low toxicity and high bioactivities (4). In particular, sulfated polysaccahrides from marine algae are known to exhibit various biological and physiological activities including antioxidant, anticoagulant, antiviral, antitumor, and anti-inflammatory activities. There is also an emerging interest in the use of naturally occurring antioxidants in foods. Honey a sweet natural product produced by honey bees form nectar, and other plant juices is also a good source of antioxidants. Antioxidant and antimicrobial properties of honey are due to presence of variety of compounds like phenolics, ascorbic acid, α-tocopherol, proline, vitamins, catalase and glucose oxidase. Extensive data is available for antioxidant properties of honeys from different origins of the world which evidenced that bioactivities of honeys vary from each other due to botanical and geographical variations (5). Due to importance of this class of compounds, assessment of antioxidant or radical-scavenging capacity has attracted increasing attention in a number of areas, which demands the availability of simple, convenient, rapid and reliable in vitro analytical methodologies. Many different methods are used to screen matrixes or to find specific antioxidants (6).

## References

[1] Troncoso N, Sierra H, Carvajal L, Delpiano P, Gunther G (2005). Fast high performance liquid chromatography and ultraviolet-visible quantification of principal phenolic antioxidants in fresh rosemary.. J Chromatogr A..

[2] Hatamnia AA, Abbaspour N, Darvishzadeh R (2013). Antioxidant activity and phenolic profile of different parts of Bene (Pistacia atlantica subsp. kurdica) fruits.. Food Chem..

[3] Thaipong K, Boonprakob U, Crosby K, Cisneros-Zevallos L, Hawkins Byrne D (2006). Comparison of ABTS, DPPH, FRAP, and ORAC assays for estimating antioxidant activity from guava fruit extracts.. J Food Compos Anal..

[4] Shao P, Chen X, Sun P (2013). In vitro antioxidant and antitumor activities of different sulfated polysaccharides isolated from three algae.. Int J Biol Macromol..

[5] Noor N, Sarfraz RA, Ali S, Shahid M (2014). Antitumour and antioxidant potential of some selected Pakistani honeys.. Food Chem..

[6] Christophe C, Belaidi FS, Launay J, Gros P, Questel E, Temple-Boyer P (2012). Elaboration of integrated microelectrodes for the detection of antioxidant species.. Sens Actuators B Chem..

